# Challenges in Managing Antipsychotic-Induced Hyperprolactinemia: A Case Study and Clinical Considerations for Aripiprazole Integration

**DOI:** 10.1192/j.eurpsy.2024.296

**Published:** 2024-08-27

**Authors:** J. Garde Gonzalez, P. Herrero Ortega, A. Oliva Lozano, M. A. Morillas Romerosa, M. N. P. Soler

**Affiliations:** Psychiatry, La Paz University Hospital, Madrid, Spain

## Abstract

**Introduction:**

Current guidelines for managing antipsychotic-induced hyperprolactinemia recommend the use of aripiprazole, either as a substitute or in combination with the primary antipsychotic. However, there have been reported cases of exacerbated psychotic symptoms when introducing aripiprazole after chronic treatment with another antipsychotic.

**Objectives:**

We present a case of a patient with unspecified schizophrenia spectrum disorder receiving amisulpride, who developed worsened psychotic symptoms following the introduction of aripiprazole to treat hyperprolactinemia. We also review this phenomenon and its clinical management in the literature.

**Methods:**

Clinical case report and brief literature review.

**Results:**

Ms. G, a 54-year-old woman diagnosed with unspecified schizophrenia spectrum disorder, was on amisulpiride 400mg/day and had remained asymptomatic for months. During a follow-up, she complained of mastalgia and had a prolactin level of 71.7 ng/mL. Following clinical guidelines, aripiprazole was added at 10mg/day. Within a month, anxiety and sleep disturbances escalated, followed by the reappearance of psychotic symptoms. Aripiprazole was discontinued, amisulpride reinstated, achieving stability. Subsequently, hyperprolactinemia was managed using metformin.

Antipsychotic-induced hyperprolactinemia is common, especially with first-generation antipsychotics, causing various symptoms in both genders. Long-term consequences may include weight gain, reduced bone density, and potentially increased breast cancer risk, among others.

Aripiprazole is an atypical antipsychotic with a partial agonist effect on dopamine D2 and D3 receptors. This means that aripiprazole will act as a functional D2-antagonist under hyperdopaminergic conditions, but a functional D2-agonist under hypodopaminergic conditions. This property of aripiprazole may contribute to counteract ‘prolactin-raising’ agents, acting as a D2 agonist, potentially reducing prolactin rise.

Chronic use of presynaptic D2/D3 antagonist (amisulpiride) might create a hypodopaminergic environment and hypersensitivity to dopamine agonists, possibly explaining worsened symptoms with aripiprazole introduction.

To prevent adverse outcomes when adding or switching to aripiprazole, gradual reduction of the previous antipsychotic (amisulpride) and high-dose aripiprazole initiation (30-40 mg/day) can help.

**Conclusions:**

Antipsychotic-induced hyperprolactinemia is a serious issue that deserves our attention. While aripiprazole is recommended by several guidelines to treat it, introducing it to patients on chronic antipsychotic treatment may lead to a worsening of psychotic symptoms. Caution is recommended when combining aripiprazole with potent D2 receptor blockers. To mitigate this effect, gradual dose reduction of the previous antipsychotic and high-dose aripiprazole initiation are crucial.

**Disclosure of Interest:**

None Declared

